# Supplementary material for: Elucidating essential kinases of endothelin signalling by logic modelling of phosphoproteomics data
Source: Mol Syst Biol. 2019 Aug 6;15(8):e8828. doi: 10.15252/msb.20198828 (PMC6683863; doi:10.15252/msb.20198828)
Supplement: Supplementary file 1 — Appendix [file MSB-15-e8828-s001.pdf]

Table of Contents

Appendix Figure S1. Randomised control network model for the UACC257 dataset

Appendix Figure S2. PKC, MEK and p70S6K inhibition confirms parts of the UACC257 network structure.

Appendix Figure S3. Only AMPK inhibition reverses EDN mediated E-Cadherin downregulation.

Appendix Table S1. Summary of the NetworKIN based prediction of cluster-enriched kinases shown in Figure 4.

Appendix Table S2. Kinase inhibitors used.

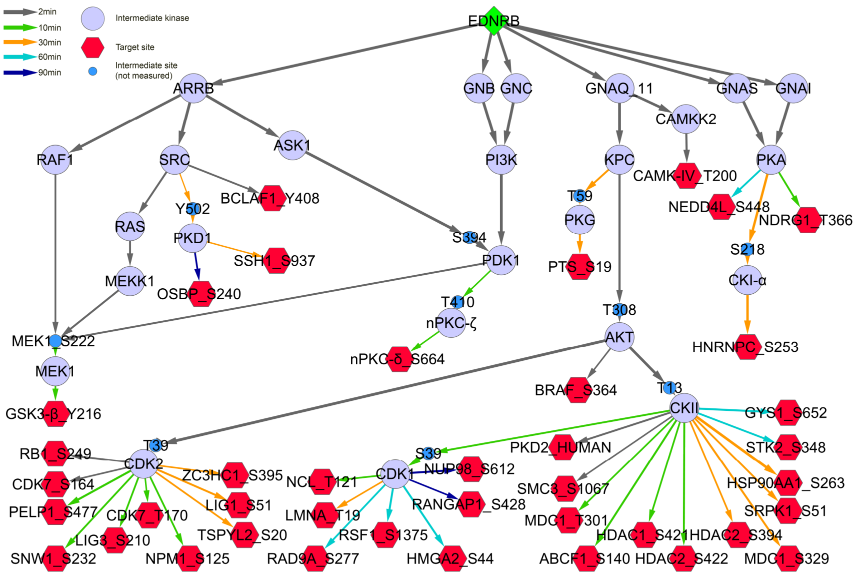

Appendix Figure S1. Randomised control network model for the UACC257 dataset

A control network model was generated based on shuffled data. Quantitative data of all EDN target sites in the UACC257 dataset (Table EV1b) were assigned randomly selected phosphosite identifiers from the non-regulated phosphosites of the same dataset. The network was generated using the same method used for Fig. 5 and Fig. EV3.

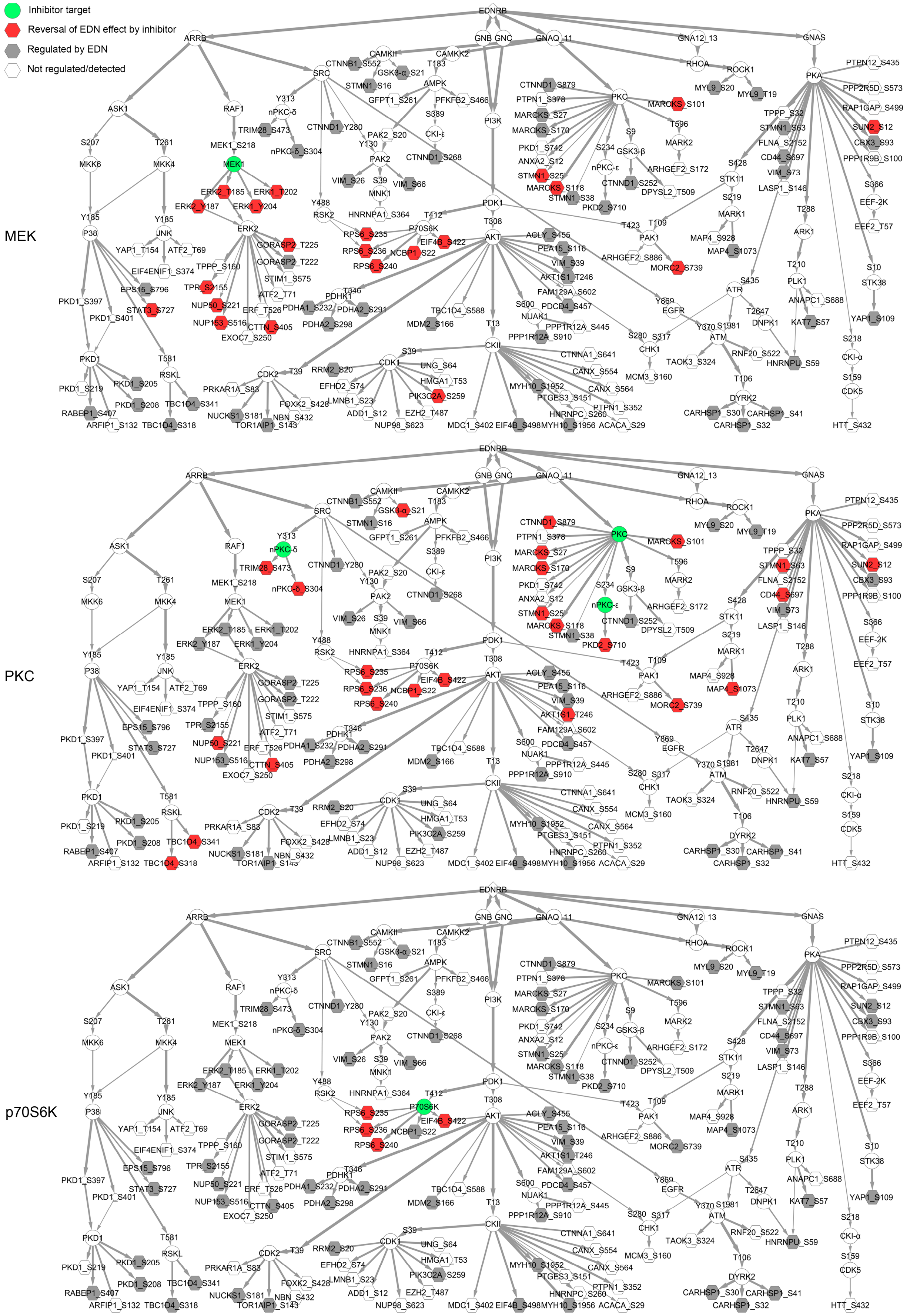
Inhibitor target
Reversal of EDN effect by inhibitor
Regulated by EDN
Not regulated/detected
MEK
PKC
p70S6K
EDNRB
ARRB
GNB GNC
GNAQ_11
GNA12_13
GNAS
CAMKII
CAMKK2
CTNNB1_S552
GSK3-α_S21
T183
SRC
STMN1_S16
AMPK
GFPT1_S261
PFKFB2_S466
S389
CKI-ε
ASK1
RAF1
Y313
nPKC-δ
TRIM28_S473
nPKC-δ_S304
CTNND1_Y280
PAK2_S20
Y130
PAK2
CTNND1_S268
VIM_S26
S39
VIM_S66
MNK1
MEK1_S218
MEK1
ERK2_T185
ERK1_T202
ERK2_Y187
ERK1_Y204
ERK2
S207
T261
MKK6
MKK4
Y185
P38
JNK
YAP1_T154
ATF2_T69
EIF4ENIF1_S374
EPS15_S796
STAT3_S727
PKD1_S397
PKD1_S401
PKD1
T581
RSKL
PKD1_S219
PKD1_S205
PKD1_S208
RABEP1_S407
ARFIP1_S132
TBC1D4_S341
TBC1D4_S318
TPPP_S160
TPR_S2155
NUP50_S221
NUP153_S516
EXOC7_S250
CTTN_S405
ERF_T526
ATF2_T71
STIM1_S575
GORASP2_T225
GORASP2_T222
Y488
RSK2
HNRNPA1_S364
T412
P70S6K
RPS6_S235
RPS6_S236
RPS6_S240
NCBP1_S22
EIF4B_S422
PDHK1
T346
PDHA1_S232
PDHA2_S291
PDHA2_S298
PI3K
PDK1
T308
AKT
ACLY_S455
PEA15_S116
VIM_S39
AKT1S1_T246
FAM129A_S602
S600
PDCD4_S457
NUAK1
PPP1R12A_S445
PPP1R12A_S910
TBC1D4_S588
MDM2_S166
T13
CKII
S39
CDK1
CDK2
T39
RRM2_S20
EFHD2_S74
UNG_S64
HMGA1_T53
PIK3C2A_S259
LMNB1_S23
ADD1_S12
EZH2_T487
NUP98_S623
FOXK2_S428
PRKAR1A_S83
NUCKS1_S181
NBN_S432
TOR1AIP1_S143
MDC1_S402
EIF4B_S498
MYH10_S1952
MYH10_S1956
PTGES3_S151
HNRNPC_S260
PTPN1_S352
CANX_S554
CANX_S564
CTNNA1_S641
ACACA_S29
MCM3_S160
CHK1
S280
S317
TAOK3_S324
CTNND1_S879
PTPN1_S378
MARCKS_S27
MARCKS_S170
PKD1_S742
ANXA2_S12
STMN1_S25
MARCKS_S118
STMN1_S38
PKD2_S710
PKC
MARCKS_S101
T596
S9
MARK2
S234
GSK3-β
nPKC-ε
CTNND1_S252
DPYSL2_T509
ARHGEF2_S172
RHOA
ROCK1
MYL9_S20
MYL9_T19
T423
T109
PAK1
MAP4_S928
MAP4_S1073
MARK1
ARHGEF2_S886
MORC2_S739
Y869
EGFR
ATR
S435
T2647
DNPK1
Y370
S1981
ATM
RNF20_S522
T106
HNRNPU_S59
DYRK2
CARHSP1_S30
CARHSP1_S41
CARHSP1_S32
STK11
S428
S219
PKA
PTPN12_S435
PPP2R5D_S573
RAP1GAP_S499
SUN2_S12
CBX3_S93
PPP1R9B_S100
TPPP_S32
STMN1_S63
FLNA_S2152
CD44_S697
VIM_S73
LASP1_S146
T288
ARK1
T210
PLK1
ANAPC1_S688
KAT7_S57
S366
EEF-2K
EEF2_T57
S10
STK38
YAP1_S109
S218
CKI-α
S159
CDK5
HTT_S432

Appendix Figure S2. PKC, MEK and p70S6K inhibition confirms parts of the UACC257 network structure.

UACC257 cells were incubated with DMSO, 100 nM MEK inhibitor (Trametinib, upper panel), 1 µM PKC inhibitor (Go 6983, middle panel) or 1 µM p70S6K inhibitor (LY2584702, lower panel) for 1 hour. Subsequently cells were mock-stimulated or treated with 100 nM EDN for 20 min and processed according to the phosphoproteomics workflow used in previous experiments (n = 3, per biological condition). Sites present in the original network models were extracted and evaluated from the resulting DDA dataset. EDN target sites were identified by comparing mock stimulated and EDN samples and reversal of EDN action was assessed by comparison of EDN / DMSO with EDN / kinase inhibitor. Q values were calculated from two-tailed unpaired t-tests followed by Benjamini Hochberg correction over all tests in the dataset. EDN and inhibitor effects were identified with cut-offs FC > 1.5 up or down and q < 0.01. Kinase targets of the inhibitors are shown in green, sites affected by EDN stimulation are shown in red if they were reversed by inhibitor treatment and in grey if they were not modulated by the kinase inhibitor. Target sites which were not detected or not affected by EDN under the conditions tested are shown in white.

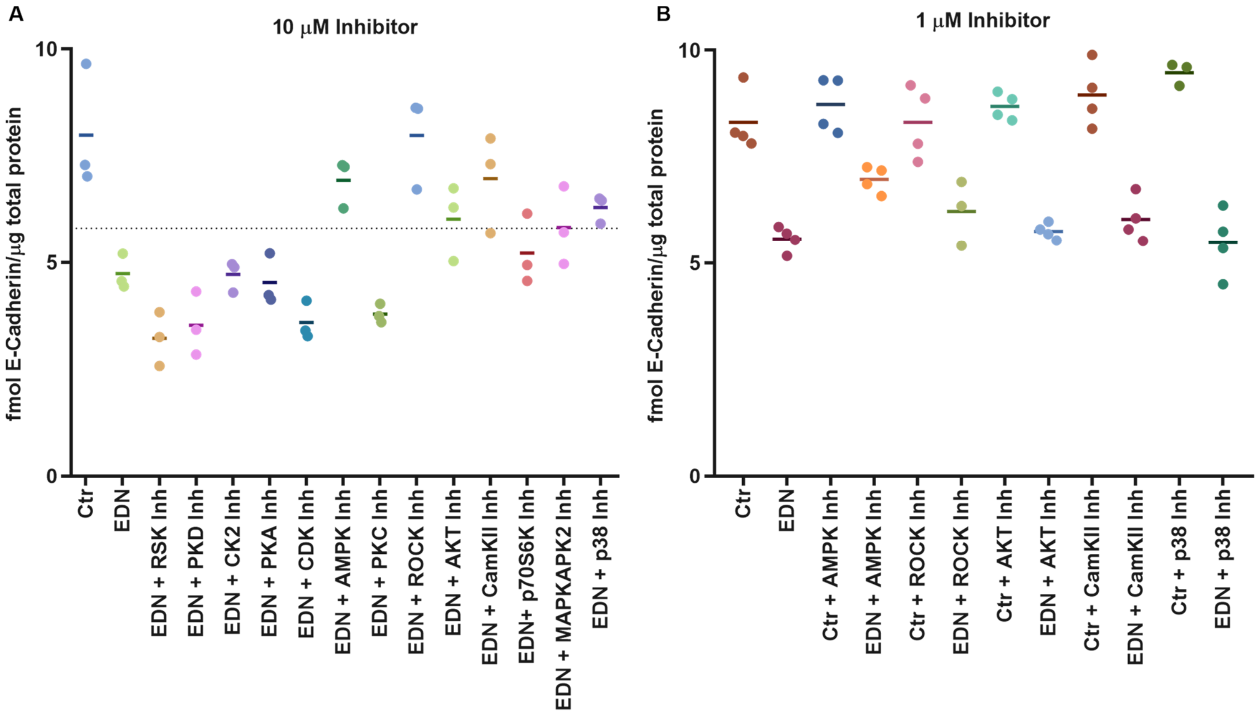

Appendix Figure S3. Only AMPK inhibition reverses EDN mediated E-Cadherin downregulation.

(**A**) Effect of 10 µM kinase inhibitor on E-Cadherin expression was evaluated in UACC257 cells stimulated with 100 nM EDN for 2 d and compared to unstimulated (Ctr) levels (n = 3) using SRM. (**B**) Effective inhibitors from **A** were tested at 1 µM after 2 d treatment. The effect of kinase inhibition on E-Cadherin expression was measured in unstimulated (Ctr) and 100 nM EDN stimulated cells (EDN) (n = 4) by SRM.

Cell Line	Cluster	Kinase	Target Sites	p-value	q-value	Odds Ratio
UACC257	C1	**CaMKIIα**	36	3.76E-07	1.81E-05	2.9
UACC257	C1	**PAK1**	24	3.00E-04	7.19E-03	2.4
UACC257	C1	**PAK2**	5	1.11E-03	1.77E-02	7.8
UACC257	C2	**CaMKIIα**	39	8.89E-09	4.71E-07	3.3
UACC257	C2	**MEK1**	6	1.23E-05	3.26E-04	16.9
UACC257	C2	**AKT**	10	2.65E-04	4.68E-03	4.4
UACC257	C2	**PAK1**	23	6.39E-04	8.47E-03	2.3
UACC257	C3	**p70S6K**	5	1.44E-05	6.34E-04	26.6
UACC257	C3	**PAK1**	20	4.08E-03	8.98E-02	2.1
UACC257	C4	**ERK1**	53	3.46E-04	1.46E-02	1.8
UACC257	C5	**GSK3-β**	17	9.38E-06	3.94E-04	4.0
A2058	C1	**CaMKIIα**	38	2.23E-16	7.35E-15	6.8
A2058	C1	**PKC-β**	15	1.92E-03	3.16E-02	2.6
A2058	C1	**PDHK1**	3	1.07E-02	7.87E-02	7.4
A2058	C1	**PKC-α**	5	1.19E-02	7.87E-02	4.0
A2058	C2	**CaMKIIα**	29	3.41E-08	1.36E-06	3.8
A2058	C2	**PKC-η**	3	1.34E-04	2.67E-03	61.9
A2058	C2	**PKD1**	3	2.59E-03	3.46E-02	13.7
A2058	C3	**AKT**	11	2.95E-06	1.24E-04	7.0
A2058	C3	**p70S6K**	5	7.29E-06	1.53E-04	30.9
A2058	C3	**CaMKIIα**	23	3.56E-03	3.74E-02	2.0
A2058	C3	**PAK1**	18	3.07E-03	3.74E-02	2.2
A2058	C3	**PAK2**	4	1.17E-02	8.18E-02	5.1
A2058	C4	**MEK1**	3	3.03E-03	1.18E-01	13.6
A2058	C5	**GSK3-β**	11	5.54E-04	1.88E-02	3.6
A2058	C6	**CDK1**	39	6.47E-10	2.20E-08	3.7

Appendix Table S1. Summary of the NetworKIN based prediction of cluster-enriched kinases shown in Figure 4.

**Kinase**	**Inhibitor**
CDKs	Dinaciclib (SCH727965)
AKT	Ipatasertib (GDC-0068)
CK2	CX–4945 (Silmitasertib)
p38	TAK-715
JNK	JNK-IN-8
PKC	Go 6983
PAK	FRAX486
RSK	BI-D1870
ROCK	GSK429286
CamKII	KN-93
MAPKAPK2	PF-3644022
PKA	2′,5′-Dideoxyadenosine
p70S6K	LY2584702
PKD1/2	CRT0066101
AMPK	Dorsomorphin
MEK	Trametinib
BRAF	Dabrafenib

Appendix Table S2. Kinase inhibitors used.
