# Supplementary material for: Elucidating essential kinases of endothelin signalling by logic modelling of phosphoproteomics data
Source: Mol Syst Biol. 2019 Aug 6;15(8):e8828. doi: 10.15252/msb.20198828 (PMC6683863; doi:10.15252/msb.20198828)
Supplement: Supplementary file 2 — Expanded View Figures PDF [file MSB-15-e8828-s002.pdf]

Expanded View Figures

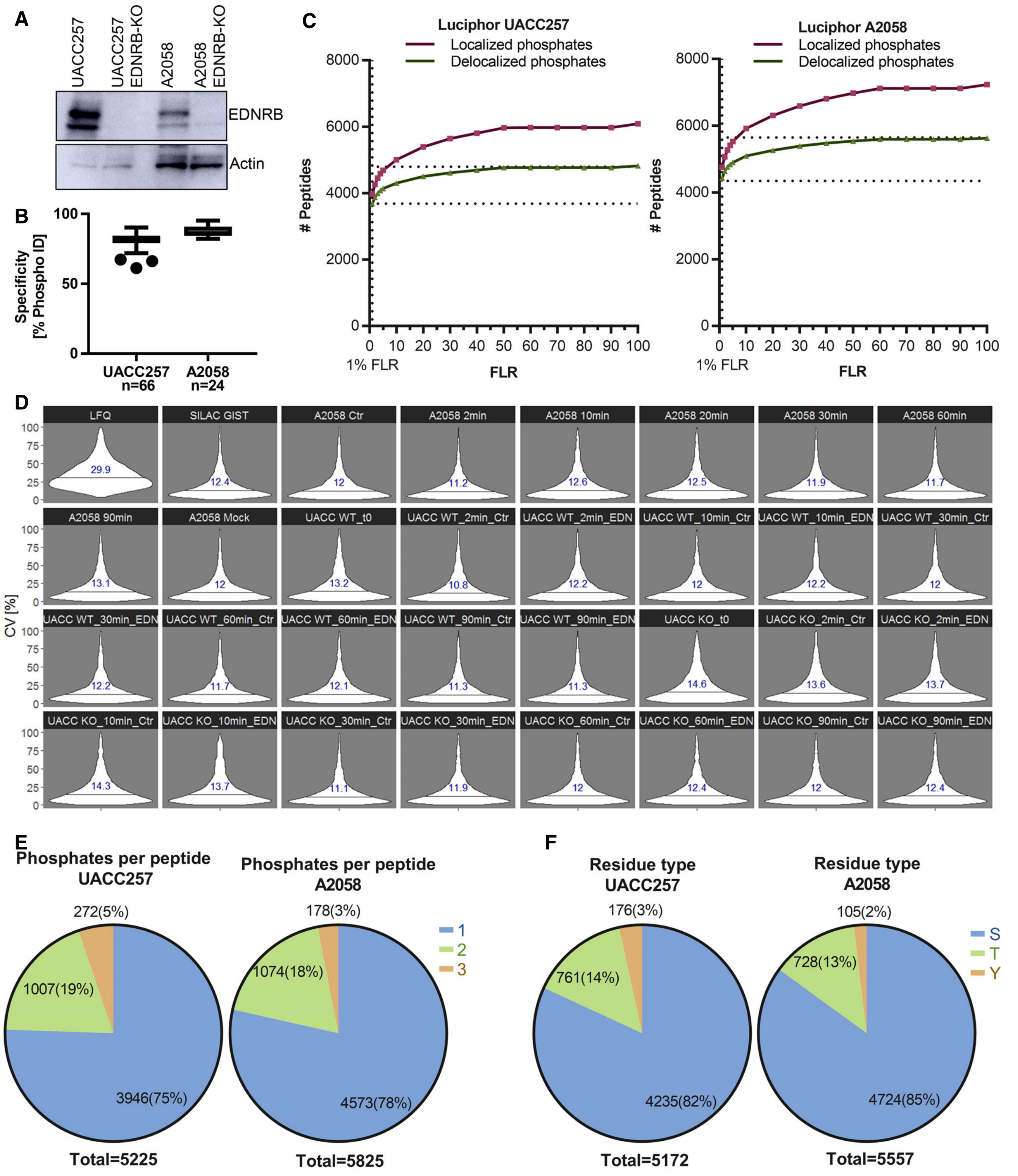

Figure EV1.

© 2019 The Authors

Figure EV1. High reproducibility of the SILAC GIST workflow and descriptive statistics of the EDN signalling phosphoproteomic data sets.

A Validation of EDNRB ablation in UACC257 and A2058. EDNRB expression in WT and EDNRB-KO cell lines was analysed by Western blot. Ten and 50 μg total protein were loaded for UACC257 and A2058, respectively.

B Specificity of TiO_2 enrichment, defined as percentage of phosphopeptide identifications over all identifications. The whiskers show 1.5*IQR (Tukey).

C LuciPHOr performance. Number of peptides with localised phosphates is compared to all phosphopeptides in the data set if phosphate positions were delocalised for different global FLR cut-offs.

D CV of phosphopeptide quantification was benchmarked in six process replicates (n = 6) of the full workflow. Peptides were quantified from the same result files using LFQ (Progenesis QI for proteomics) or the Skyline SILAC GIST workflow. CV violin plots for benchmarking samples and each triplicate in the UACC257 and A2058 data sets are shown. Lines and numbers indicate the medians for each group.

E Proportions of singly, doubly and triply phosphorylated peptides for each cell line.

F Proportions of phosphorylated amino acid residues for each data set.

Figure EV2. EDN stimulation leads to altered phosphorylation patterns in UACC257 and A2058 cells.

A Hierarchical clustering of phosphopeptide MS1 intensity for UACC257 separates samples according to the presence and activation of the EDNRB receptor. Clustering was performed on the UACC257 data set in R following $\log_2$ transformation of normalised MS1 intensities, imputation of missing values with the imputeLCMD R package and z-score transformation.

B PCA of the A2058 data set. Phosphopeptide MS1 intensities in A2058 samples were analysed by PCA using R. Colours indicate experimental groups, and shading is mapped to time.

C Examples kinetics of known (ERK1 T202/Y204) and novel phosphosites (MEK2 S23 & ROCK2 S1134/S1137) on relevant kinases in UACC257 (top) and A2058 (bottom) cells from phosphoproteomic data sets. Data are represented as mean ± SD. *q < 0.1. Two sided unpaired t-tests followed by Benjamini–Hochberg multiple testing correction (n = 3).

© 2019 The Authors

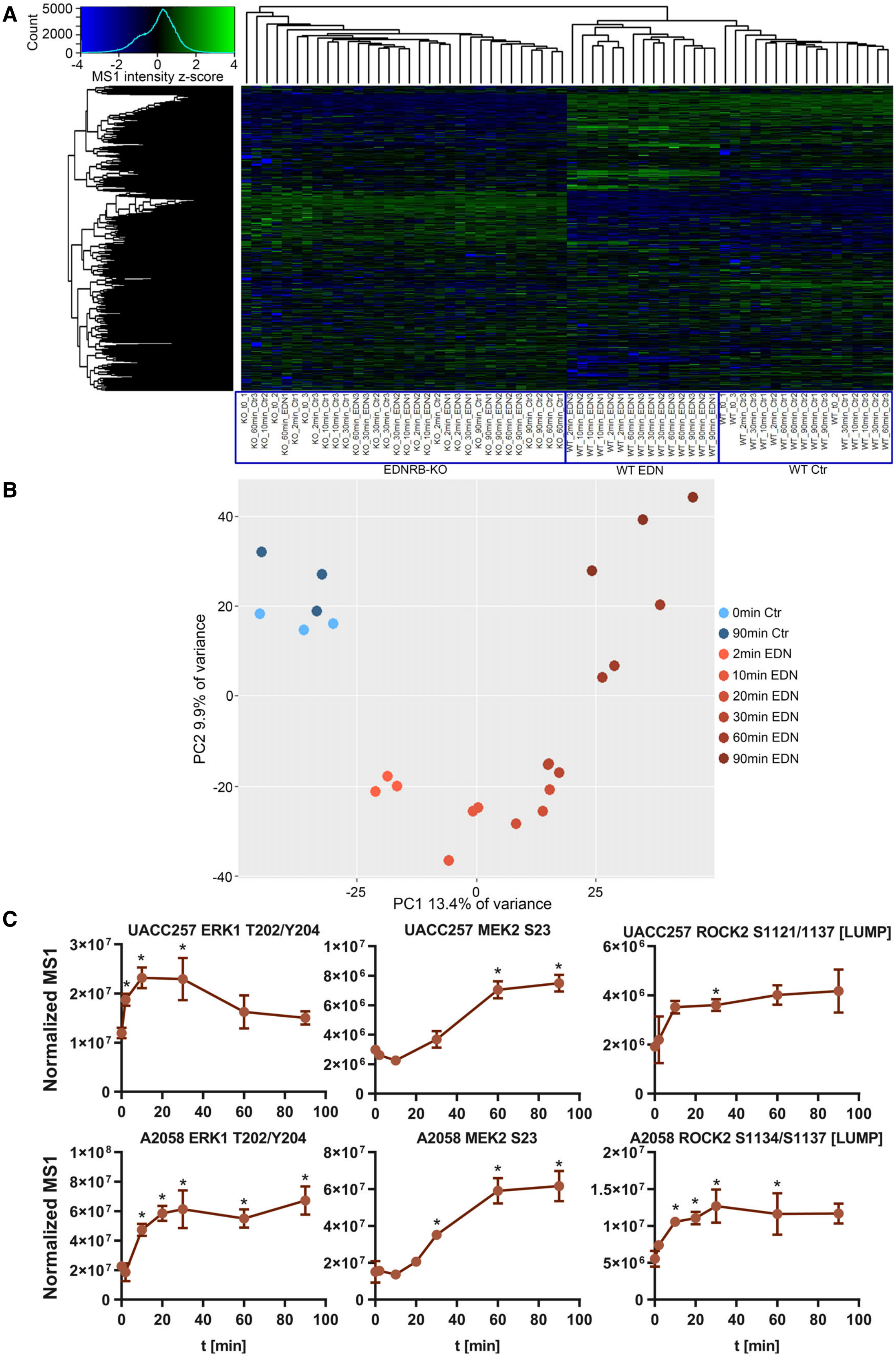

Figure EV2.

 © 2019 The Authors

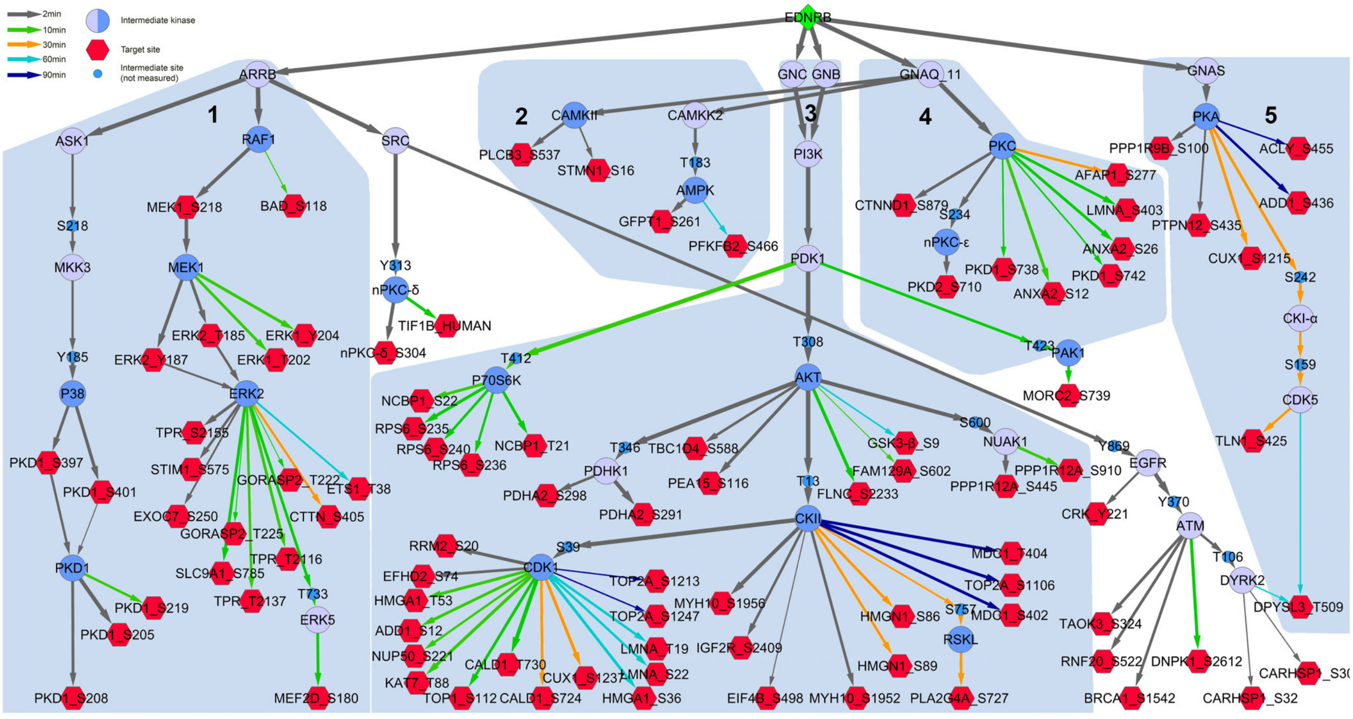

Figure EV3. Prior knowledge-based time-resolved network model of EDN signalling in A2058 cells.
Construction of the network is described in detail in Materials and Methods. EDNRB (green diamond) was connected to its target sites (red hexagons) through intermediary kinases (blue circles) in a time-resolved variant of the PHONEMeS approach. Central kinases, which were also identified by kinase activation prediction, are shown as intermediary kinases with dark blue shading. Edge weights were assigned by downsampling the network 100 times. Entry time point was defined as the point at which edge weight reached 20 and is shown as edge colour. The network is divided into five modules, indicated as light blue outlines and labelled 1–5. Common names are shown for kinases, and primary gene names are shown for all other proteins.

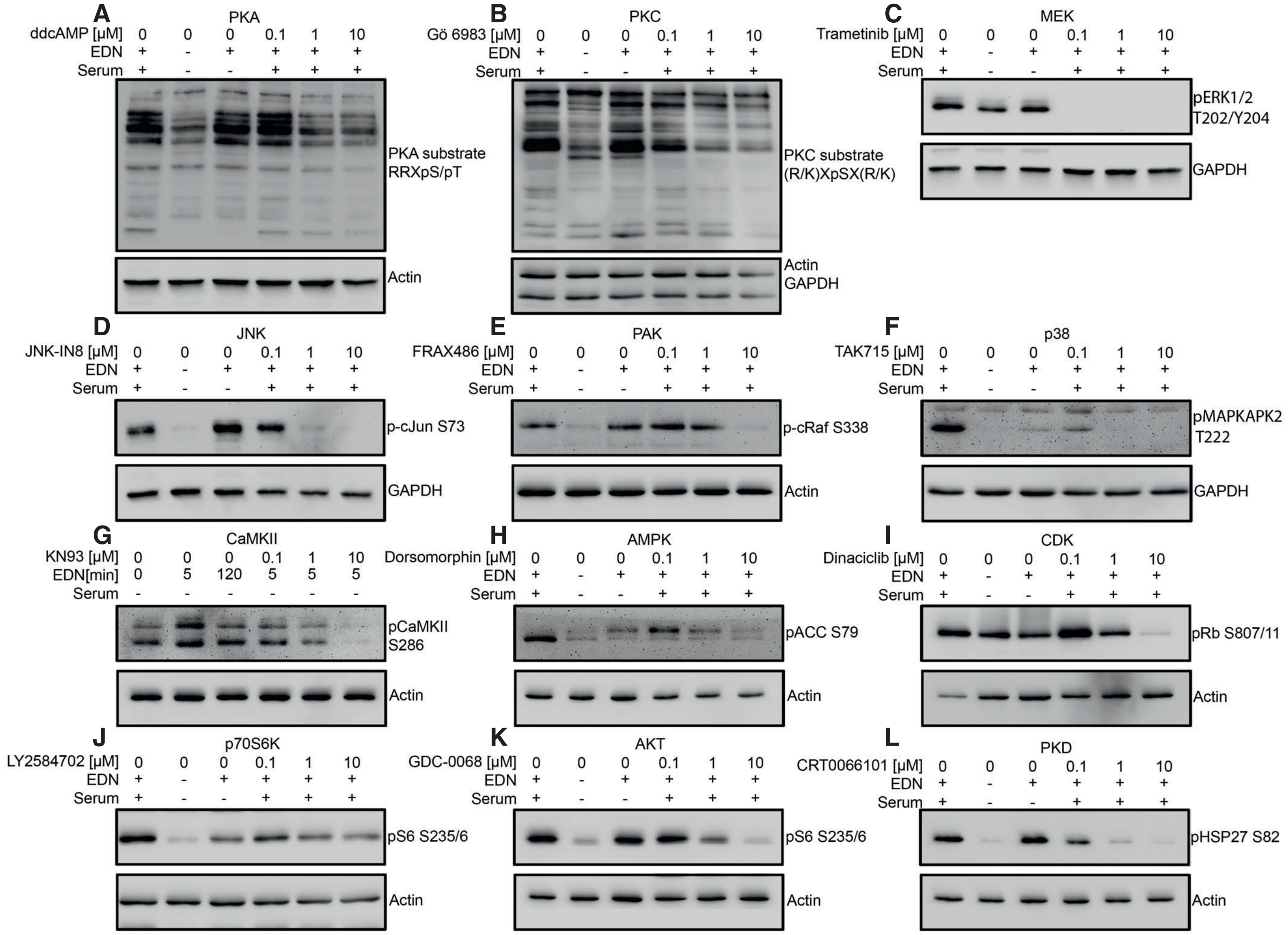

Figure EV4. Validation of kinase inhibitor efficacy.

A–L Extended view of the Western blots shown in Fig 6. In addition to serum starved UACC257 cells treated with PBS or 100 nM EDN, cells in full medium (10% FCS) were treated with EDN + DMSO or increasing (0.1, 1, 10 μM) concentrations of an inhibitor for the kinase under study and analysed by Western blot. Phosphorylated kinase substrates were detected with phosphospecific antibodies. Representative example of two independent experiments.

Figure EV5. Extended data and additional controls for the cell migration validation experiments.

- A Average changes in cell free area (n = 3) for all inhibitors tested in the screen presented in Fig 7 are shown as a heat map. X = not done.
- B EDN-mediated scratch closure is not caused by proliferation. Proliferation was measured as a function of EDN treatment over 3 days using a BrdU ELISA assay in a separate experiment (n = 3). Error bars show SD.
- C Kinase inhibitor treatment does not influence cell viability. At the end of the scratch experiments, UACC257 cell viability was measured using the MTT method. Values were normalised to Ctr (DMSO) for each inhibitor (n = 3). Error bars show SD.

▶

 © 2019 The Authors

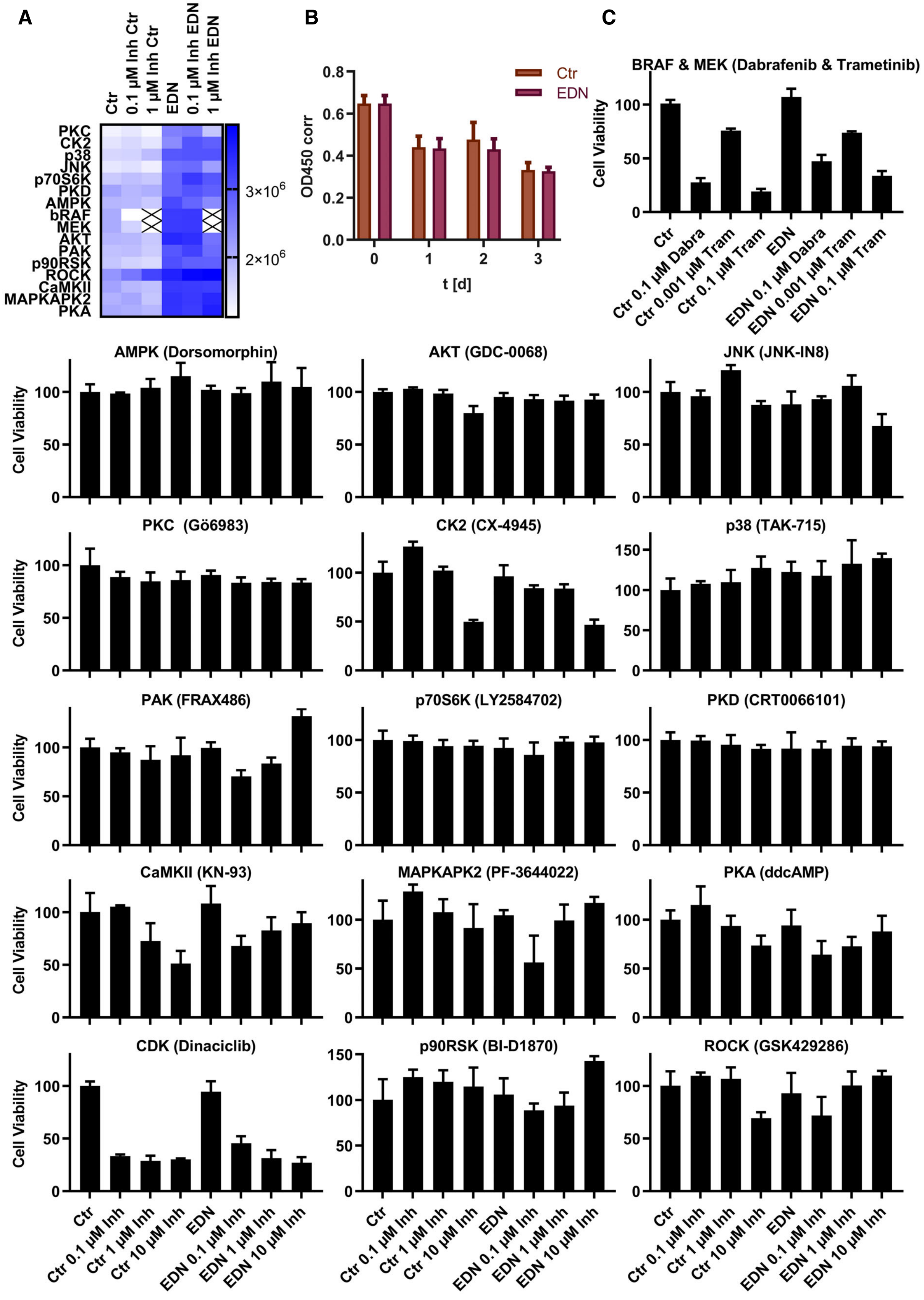
A
Ctr
0.1 µM Inh Ctr
1 µM Inh Ctr
EDN
0.1 µM Inh EDN
1 µM Inh EDN
PKC
CK2
p38
JNK
p70S6K
PKD
AMPK
bRAF
MEK
AKT
PAK
p90RSK
ROCK
CaMKII
MAPKAPK2
PKA
3×10^6
2×10^6
B
Ctr
EDN
OD450 corr
t [d]
C
BRAF & MEK (Dabrafenib & Trametinib)
Cell Viability
Ctr
Ctr 0.1 µM Dabra
Ctr 0.001 µM Tram
Ctr 0.1 µM Tram
EDN
EDN 0.1 µM Dabra
EDN 0.001 µM Tram
EDN 0.1 µM Tram
AMPK (Dorsomorphin)
AKT (GDC-0068)
JNK (JNK-IN8)
PKC (Gö6983)
CK2 (CX-4945)
p38 (TAK-715)
PAK (FRAX486)
p70S6K (LY2584702)
PKD (CRT0066101)
CaMKII (KN-93)
MAPKAPK2 (PF-3644022)
PKA (ddcAMP)
CDK (Dinaciclib)
p90RSK (BI-D1870)
ROCK (GSK429286)
Ctr
Ctr 0.1 µM Inh
Ctr 1 µM Inh
Ctr 10 µM Inh
EDN
EDN 0.1 µM Inh
EDN 1 µM Inh
EDN 10 µM Inh

Figure EV5.
